# Generation and transplantation of reprogrammed human neurons in the brain using 3D microtopographic scaffolds

**DOI:** 10.1038/ncomms10862

**Published:** 2016-03-17

**Authors:** Aaron L. Carlson, Neal K. Bennett, Nicola L. Francis, Apoorva Halikere, Stephen Clarke, Jennifer C. Moore, Ronald P. Hart, Kenneth Paradiso, Marius Wernig, Joachim Kohn, Zhiping P. Pang, Prabhas V. Moghe

**Affiliations:** 1 Department of Biomedical Engineering, Rutgers University, 599 Taylor Road, Piscataway, New Jersey 08854, USA; 2 Department of Neuroscience and Cell Biology, Rutgers Robert Wood Johnson Medical School, 89 French Street, New Brunswick, New Jersey 08854, USA; 3 Child Health Institute of New Jersey, Rutgers Robert Wood Johnson Medical School, 89 French Street, New Brunswick, New Jersey 08854, USA; 4 Department of Cell Biology and Neuroscience, Rutgers University, 604 Allison Road, Piscataway, New Jersey 08854, USA; 5 Human Genetics Institute of New Jersey, 145 Bevier Road, Piscataway, New Jersey 08854, USA; 6 Institute for Stem Cell Biology and Regenerative Medicine, Stanford University School of Medicine, Stanford, California 94305, USA; 7 Department of Chemistry and Chemical Biology, New Jersey Center for Biomaterials, 145 Bevier Road, Piscataway, New Jersey 08854, USA; 8 Department of Chemical and Biochemical Engineering, Rutgers University, 98 Brett Road, Piscataway, New Jersey 08854, USA

## Abstract

Cell replacement therapy with human pluripotent stem cell-derived neurons has the potential to ameliorate neurodegenerative dysfunction and central nervous system injuries, but reprogrammed neurons are dissociated and spatially disorganized during transplantation, rendering poor cell survival, functionality and engraftment *in vivo*. Here, we present the design of three-dimensional (3D) microtopographic scaffolds, using tunable electrospun microfibrous polymeric substrates that promote *in situ* stem cell neuronal reprogramming, neural network establishment and support neuronal engraftment into the brain. Scaffold-supported, reprogrammed neuronal networks were successfully grafted into organotypic hippocampal brain slices, showing an ∼3.5-fold improvement in neurite outgrowth and increased action potential firing relative to injected isolated cells. Transplantation of scaffold-supported neuronal networks into mouse brain striatum improved survival ∼38-fold at the injection site relative to injected isolated cells, and allowed delivery of multiple neuronal subtypes. Thus, 3D microscale biomaterials represent a promising platform for the transplantation of therapeutic human neurons with broad neuro-regenerative relevance.

## Abstract (web summary)

Human pluripotent stem cell derived neurons have the potential for cell replacement therapy for brain injury and disease but problems on transplantation need to be overcome. Here, the authors use a microtopographic scaffold to graft neurons into both hippocampal organoids and the mouse brain striatum.

## Graphical abstract

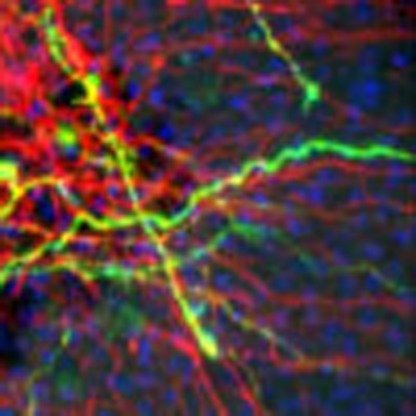

Neurodegenerative diseases and traumatic brain injuries result in a loss of functional neurons in the central nervous system (CNS) and are responsible for substantial deterioration in quality of life. Although cell transplantation therapies have shown some promise towards functional recovery in animal models, the efficacy of these therapies has been limited by poor cell survival rates^1^,^2^,^3^. Human induced pluripotent stem (iPS) cells have recently emerged as a promising renewable source of expandable patient-specific cells that can be used to generate human neurons^4^,^5^,^6^. These iPS cell-derived neurons are a potentially invaluable tool as a cell source for the *in vivo* treatment of neurodegenerative diseases and traumatic CNS injury^7^,^8^.

While many neuronal differentiation protocols have been established, a robust protocol was recently advanced for the accelerated production of human neuronal cells from iPS cells, called induced neuronal (iN) cells, by the direct conversion of iPS cells using ectopic expression of sets (*Brn2*, *Ascl1*, *Myt1l*) or single transcription factors (*Ascl1*, *NeuroD1* or *Ngn2*) (refs 9, 10, 11, 12). Direct conversion yields improved conversion efficiency and accelerates maturation relative to standard differentiation protocols. However, effective approaches to generate these cells in three-dimensional (3D) configurations and to deliver these cells therapeutically *in vivo* have yet to be established. Cell replacement therapies of matured neurons in the brain have conventionally been limited to injection of dissociated cells^2^,^13^. An alternative approach using biomaterial scaffolds can provide structural support to cells during transplantation, which could improve cell engraftment and survival. In addition, many types of cells behave differently when cultured in two-dimensional (2D) versus 3D substrates^14^,^15^, leading to the development of 3D biomaterials that better mimic aspects of the *in vivo* cellular microenvironment^16^. In particular, microscale fibrous substrates enhance several neural cell behaviours *in vitro*, including neurite outgrowth^17^, neuronal maturation^18^ and neuronal differentiation. These results motivate the interfacing of microscale fibrous scaffolds with human induced neuronal cells for *in vitro* and *in vivo* applications. The concept of using 3D biomaterials to support transplantation has been used with synthetic hydrogels^19^, microparticles^20^ and natural protein matrices^21^,^22^ to transplant neural progenitor cells into the brain, however, these sorts of scaffolds are not conducive to extended culture or maturation of cells *in vitro*, and yield limited control of the cell microenvironment once transplanted.

In this study, we advance the concept of designing electrospun synthetic polymer fibres to support the neuronal reprogramming of iPS cells within a 3D environment via the ectopic expression of *NeuroD1*. We demonstrated that fine-tuning the fibrous architecture enhances neuronal maturation and functionality *in vitro*, and also reduces the population of residual unconverted cells. Finally, we investigated the role of micron-scale fibrous scaffolds as injectable transplantation vehicles for adherent networks of iNs, and their ability to enhance the survival and engraftment of these neurons in murine brain tissue *ex vivo* and *in vivo*.

## Results

### Induction of neurons via single defined transcription factors

We initially identified that of the four transcription factors (TFs), that is, *Brn2, Ascl1, Myt1L* and *NeuroD1*, used by Pang *et al*.^12^ to reprogram human fibroblasts to neurons, only *Ascl1* and *NeuroD1* could individually induce neuronal conversion of iPS cells, as also reported elsewhere^10^. Early neurons induced by *NeuroD1* expression exhibited complex neuronal morphologies and express mature neuronal markers, so all subsequent studies were done using *NeuroD1* as the single TF for generating iN cells. We transduced human iPS cells with lentiviruses encoding tetracycline-inducible, tetOn-*NeuroD1*, rtTA, and, in selected cases, tetOn-*EGFP*, and maintained them as undifferentiated iPS cells in mTeSR medium for multiple passages in the absence of doxycycline (*dox*; Supplementary Fig. 1a). We refer to these cells as ‘RN-iPS' cells, since these cells were transduced with rtTA and NeuroD1. RN-iPS cells maintain expression of pluripotency markers SSEA-4 and Oct-4 over multiple passages in the absence of *dox*, indicating that they remain undifferentiated after transduction (Supplementary Fig. 1a).

To assess neuronal induction from RN-iPS cells, dense cultures of RN-iPS cells were seeded and *dox* was added 24 h after plating (Supplementary Fig. 1b). Early stage iN cells expressed neuronal marker βIII-tubulin and residual undifferentiated iPS cells expressed Oct-4 (Supplementary Fig. 1c). *Dox* addition rapidly induces the loss of undifferentiated morphology and the acquisition of bipolar, early neuronal morphologies in a subset of cells within 48 h (Supplementary Fig. 1c–e). *Dox* also rapidly induces *EGFP* expression in control cells transduced with tetOn-*EGFP*. By contrast, cells transduced with only tetOn-*EGFP* and rtTA (lacking tetOn-*NeuroD1*) show strong GFP fluorescence but maintain undifferentiated hPSC morphology upon *dox* addition (Supplementary Fig. 1e).

**Figure 1:**
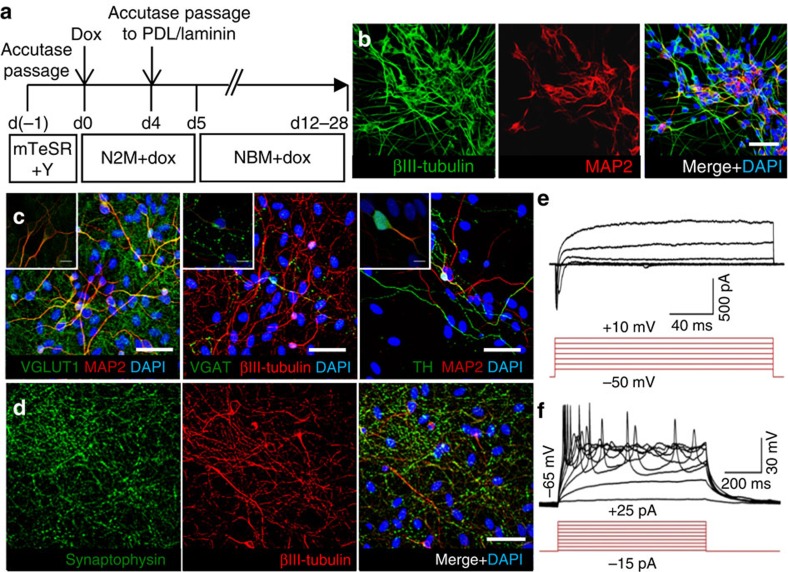
Characterization of neuronal conversion and maturation in human induced-neuronal cells. (**a**) Schematic of long-term iN reprogramming to functional neurons. (**b**) After 12 days of conversion, iN cells express βIII-tubulin and MAP2. (**c**) Further differentiation on glial cells for 28 days allows specification of several neuronal subtypes, including vesicular GABA transporter-expressing GABAergic neurons and tyrosine hydroxylase-expressing dopaminergic neurons, in addition to the predominantly glutamate vescular transporter-expressing glutamatergic neurons. (**d**) Day 28 iN cells also extensively express mature neuronal makers such as the synaptic vesicle protein synaptophysin. (**e**) Whole-cell current recordings demonstrate that iN cells after 14 days culture in the absence of glia are predominantly electrically active with functional voltage-dependent Na^+^ channels, as well as voltage-dependent K^+^ channels, and (**f**) fire repetitive induced-action potentials (*n*=27/27). Scale bar, 50 μm. Inset: Scale bar, 10 μm.

Despite the rapid, *dox*-induced neuronal conversion of a subset of RN-iPS cells, the remaining undifferentiated cells proliferated and rapidly overtook iN cultures. To address this, cells were replated 4 days post *dox* treatment, which established more uniform human neuronal cultures and eliminated many undifferentiated cells (Fig. 1a). The replating process may disrupt the cell–cell contacts necessary for undifferentiated iPS cell survival. After replating, enriched iN cells could be maintained for 4 weeks or longer before characterization.

As expected, human iN cells robustly express βIII-tubulin and microtubule-associated protein 2 (MAP2) by day 12–14 after *dox* addition (Fig. 1b). To identify the neuronal subtypes generated by this procedure, iN cells were replated 4 days after *dox* addition onto glial cell monolayers as reported elsewhere^23^. Immunocytochemistry on 28 day iNs post replating revealed that most cells expressed glutamate vesicular transporter VGLUT1, indicating that these cells are predominantly excitatory glutamatergic neurons (Fig. 1c). Occasional cells expressing markers of other neuronal subtypes were also observed, including inhibitory GABAergic neurons (expressing vesicular GABA transporter) and dopaminergic neurons (expressing tyrosine hydroxylase; Fig. 1c). Most cells also robustly expressed the pre-synaptic protein synaptophysin (Fig. 1d). Finally, patch-clamp electrophysiology demonstrated that iN cells are electrically active and express functional voltage-dependent Na^+^ channels as well as voltage-dependent K^+^ channels as revealed by whole-cell current recordings (Fig. 1e). Strikingly, these human iN cells were capable of firing repetitive action potentials (Fig. 1f). Taken together, these data indicate the robust generation of functional neuronal cells via *NeuroD1* overexpression.

### Generation of functional neurons on 3D electrospun fibres

**Figure 2:**
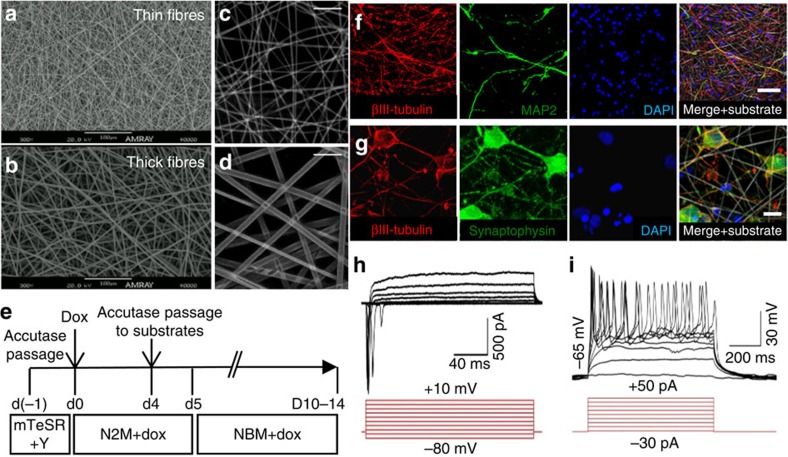
Characterization of electrospun polymer fibres and validation for support of iN differentiation. (**a**,**b**) Scanning electron microscopy and (**c**,**d**) reflectance images of 2D fibrous and 3D electrospun pDTEc fibres, with substantially variable fibre architectures and porosities that respectively do not allow and allow cellular infiltration. (**e**) Schematic of RN-iPS cell reprogramming on 3D electrospun fibres. (**f**,**g**) RN-iPS reprogramming was carried out on 3D electrospun fibres, demonstrating that generation of βIII-tubulin and MAP2 positive iN cells on 3D electrospun fibres after 12 days proceeds similarly to 2D controls shown in Fig. 1. (**h**) Whole-cell current recordings demonstrate that iN cells cultured for 10 days on 3D electrospun fibres, 14 days total were predominantly electrically active with functional voltage-dependent Na^+^ channels, as well as voltage-dependent K^+^ channels, and (**i**) fire repetitive induced action potentials (*n*=18/20). Scale bar, 50 μm (**c**–**d**,**f**). Scale bar, 10 μm (**g**).

Next, we investigated whether *NeuroD1* expression would similarly induce neuronal conversion and maturation within model 3D electrospun substrates. We constructed fibrous substrates by electrospinning poly(desaminotyrosyl tyrosine ethyl ester carbonate) (pDTEc), into two architectures, which will be referred to as ‘thin' and ‘thick' fibre substrates with average fibre diameters of 1.25±0.05 μm and 3.23±0.06 μm, respectively^24^ (Fig. 2a–d). pDTEc is the lead candidate polymer from a combinatorial library of tyrosine-derived polycarbonates^25^, as it effectively supports pluripotent stem cell culture when fabricated into microscale fibrous substrates^24^, and is biocompatible^26^. Similarly, our results with 3D polymeric substrates compared with 2D polymeric substrates suggest that the fibrous architecture governs the longer term cellular behaviours observed in contrast to the polymer composition that plays a role on early interfacial phenomena. The thick fibre scaffolds are volumetrically permeable to cellular infiltration, whereas the thin fibre scaffolds are relatively impermeable, due to decreased void space between fibres. Without additional material modifications, it is difficult to produce equal fibre sizes with variable porosity, as both properties are simultaneously modulated when altering electrospinning parameters. We hypothesize that cell permeable, thick fibre substrates will support improved iN maturation and functionality by promoting enhanced 3D organization and cell–cell contacts relative to less permeable, thin fibre substrates and 2D controls.

Human iN cells were generated within 3D constructs by treating RN-iPS cells, cultured on 2D tissue-culture plates, with *dox* for 4 days, followed by replating onto 3D electrospun substrates or 2D controls, according to the time course schematic shown in Fig. 2e. Human iN cells on 3D electrospun fibres showed complex morphology with extensive neurite outgrowth and expressed βIII-tubulin, MAP2 and synaptophysin after 12 days of reprogramming, similarly to 2D cultures (Fig. 2f,g). In addition, electrophysiological recordings revealed iN cells in electrospun substrates fired action potentials, demonstrating the derivation of functional iN cells (Fig. 2h,i).

### Effect of 3D scaffold architecture on human iN conversion

**Figure 3:**
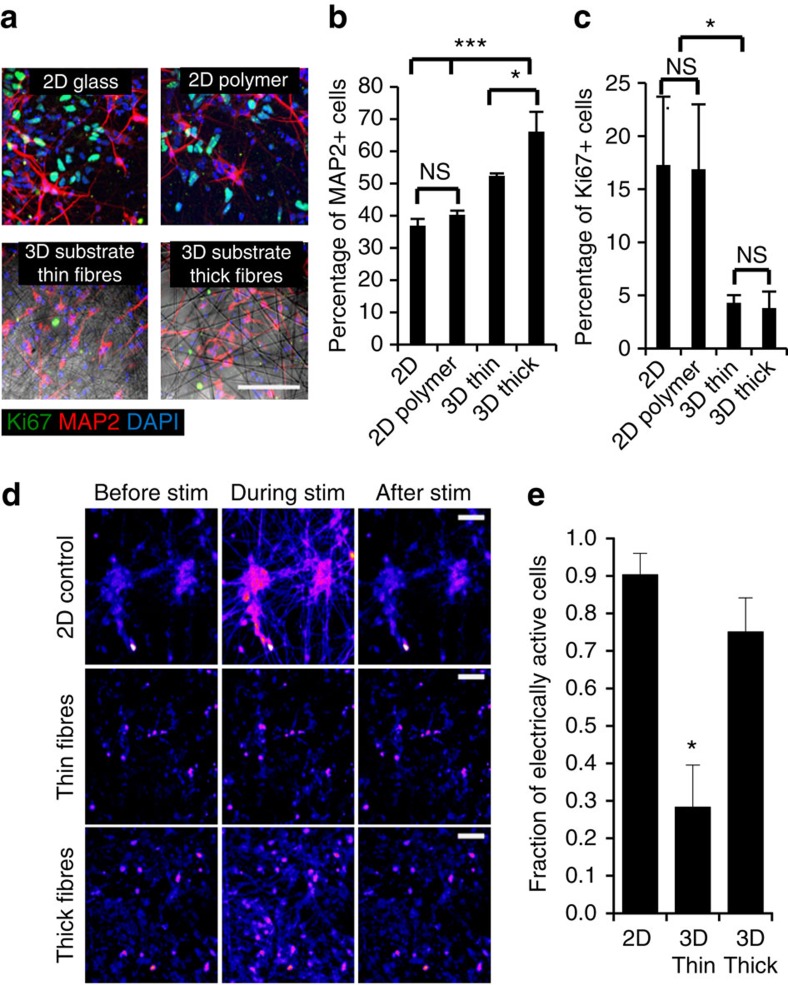
Comparison of neuronal selection and maturation in 2D and 3D substrates. (**a**) Human iN populations robustly express MAP2 in 2D and 3D conditions, while populations of unconverted, proliferative Ki67-expressing iPS cells persist in iN populations plated in 2D conditions. Scale bar, 100 μm. (**b**) Quantification reveals an enhancement of maturation as assessed by MAP2 expression in 3D electrospun thick fibres relative to thin fibres, as well as in both 3D fibrous conditions relative to 2D conditions. *n*=3, *P<0.05, ***P<0.001 by one-way ANOVA. (**c**) Quantification reveals an enhancement of neuronal selection, as assessed by Ki67 expression, fewer residual proliferative cells remained in iNs replated into 3D fibrous conditions relative to 2D conditions. Scale bar, 100 μm. *n*=3; *P<0.05, by one-way ANOVA. (**d**) Heat map images from calcium recordings of human iN cells before (left), during (middle) and after field electrical stimulation). Scale bar, 75 μm. (**e**) Quantification of the fraction of cells that respond to electrical stimulation with a substantial increase in fluorescence intensity reveals highly active cell populations in 2D and 3D thick fibre substrates relative to cells in the thin fibre substrates. *n*=3; *P<0.05 by one-way ANOVA, all error bars presented as mean±1 s.d.

Next, we examined whether the fibre architecture could be tuned to enhance human iN maturation, as we and others have shown that geometric cues can influence both human iPS cell and neural cell behaviours^24^,^27^. RN-iPS cells were treated with *dox* for 4 days, then replated onto 3D fibrous substrates or 2D controls, including 2D polymer-coated controls, for an additional 8 days of culture. Immunocytochemistry for proliferation marker Ki67 and Oct-4 revealed that significantly more proliferative and pluripotent cells were retained when iN cells were replated onto 2D substrates compared with 3D fibrous substrates (*P*<0.05, one-way ANOVA; Fig. 3a,c, Supplementary Figs 2,3). This suggests that the fibrous architectures may be able to selectively reduce the presence of residual proliferative and pluripotent iPS cells.

Human iN cells in all conditions expressed extensive βIII-tubulin-positive processes, along with robust MAP2 expression (Fig. 3a, Supplementary Fig. 2). Significantly greater numbers of human iN cells expressed MAP2 in thick fibre substrates relative to 2D controls (*P*<0.0001, one-way ANOVA) and thin fibre substrates (*P*<0.05, one-way ANOVA), indicating accelerated maturation (Fig. 3b). qRT–PCR also revealed increasing trends in expression of several neuronal genes in 3D substrates relative to 2D controls, including βIII-tubulin, MAP2, synapsin 1 and VGLUT1, though not statistically significant (Supplementary Fig. 4). Most importantly, calcium imaging to identify the fraction of cells that respond to a field electrical stimulation indicated that thick fibre substrates yielded a high degree of activity, namely, >70% electrically active cells by day 12 of culture (Fig. 3d–e), which was significantly greater than those on the thin fibre substrates (*P*<0.05, one-way ANOVA). Inhibition of E-cadherin-dependent cell–cell contacts markedly reduced neuronal outgrowth in 2D, and decreased activity measured by calcium imaging for iNs on 2D and 3D thick fibre substrates but not thin fibre substrates (Supplementary Fig. 5). This indicates that 3D microfibrous architectures establish neuronal networks with enhanced cell–cell contacts and influence both functional and phenotypic maturity of iN cellular networks.

### Microscale scaffolds support outgrowth and survival in brain

Next, transplantable constructs were designed to deliver human iN cells into the brain for regenerative therapies. The large mats of electrospun fibres (0.3–2 cm diameter discs) conventionally fabricated for *in vitro* studies cannot be easily transplanted into the CNS. To allow for injection *in vivo*, 100 μm square ‘microscale scaffolds' that could be injected through a 21-gauge needle were created by downscaling thick fibre electrospun substrates with a Vibratome. Human iN cells were seeded in suspension onto microscale scaffolds analogous to our previous studies, which resulted in efficient population of microscale scaffolds with iN cells. Cells in microscale scaffolds matured into βIII-tubulin and MAP2-expressing neuronal cells, similarly to cells on macroscale fibrous substrates (Supplementary Fig. 6). The average number of live human iN cells in each scaffold was 83±13 (*n*=19; Supplementary Fig. 7).

**Figure 4:**
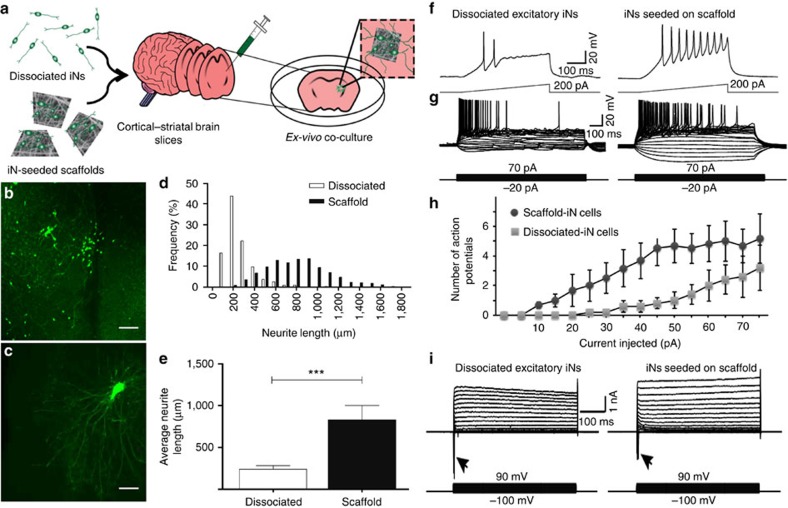
iNs supported by scaffolds support outgrowth and survival *ex vivo*. (**a**) Hundred-micrometre edge scaffolds were cut from electrospun fibres before seeding with iNs, followed by injection onto *ex vivo* cultured mouse pup brain slices. (**b**–**e**) Neurite length was found to be significantly enhanced in transplanted scaffold-supported iNs when comparing dissociated GFP-labelled iNs (**b**) with iNs seeded on scaffolds (**c**) injected onto mouse *ex vivo* brain slices (*n*=8 brain slices for each transplantation mode). (**f**–**h**) Both scaffold-supported iNs (*n*=6) and dissociated iNs (*n*=5) fire action potentials in response to current injection 14 days after transplantation onto mouse *ex vivo* brain slices, however, scaffold-supported iNs displayed enhanced excitability relative to dissociated iNs. Both modes of transplanted iNs were observed to have mature sodium channel expression (**i**). ***P<0.0001 by one-way ANOVA. Scale bar, 20 μm. All error bars presented as mean±1 s.d.

The ability of microscale electrospun scaffolds to promote human iN cell survival and engraftment was first assessed using an *ex vivo* model consisting of organotypic hippocampal slice cultures from *NOD-SCID IL2Rγc* null mice (Fig. 4a). Human iN cells on 4–5 microscale scaffolds were injected into hippocampal slices, alongside equivalent numbers of dissociated cells on paired slices, and engraftment and functionality was assessed. Immunocytochemistry revealed that 3 days after transplantation, injected scaffold-supported iN cells had average neurite lengths of 831±169 μm, which was significantly greater (*P*<0.0001, one-way ANOVA) than those of injected dissociated iNs, which had neurite lengths of 241±42 μm (Fig. 4b–e). Electrophysiological recordings from human iN cells after 3 weeks indicated that both dissociated and scaffold-supported iN cells fire action potentials in response to current injection (Fig. 4f,g), with scaffold-seeded iN cells displaying enhanced excitability in response to injected currents (Fig. 4h). In addition, both modes of transplanted cells had mature Na^+^ channel expression (Fig. 4i). This suggests that microscale scaffolds enhance engraftment and functionality of transplanted iN cells.

**Figure 5:**
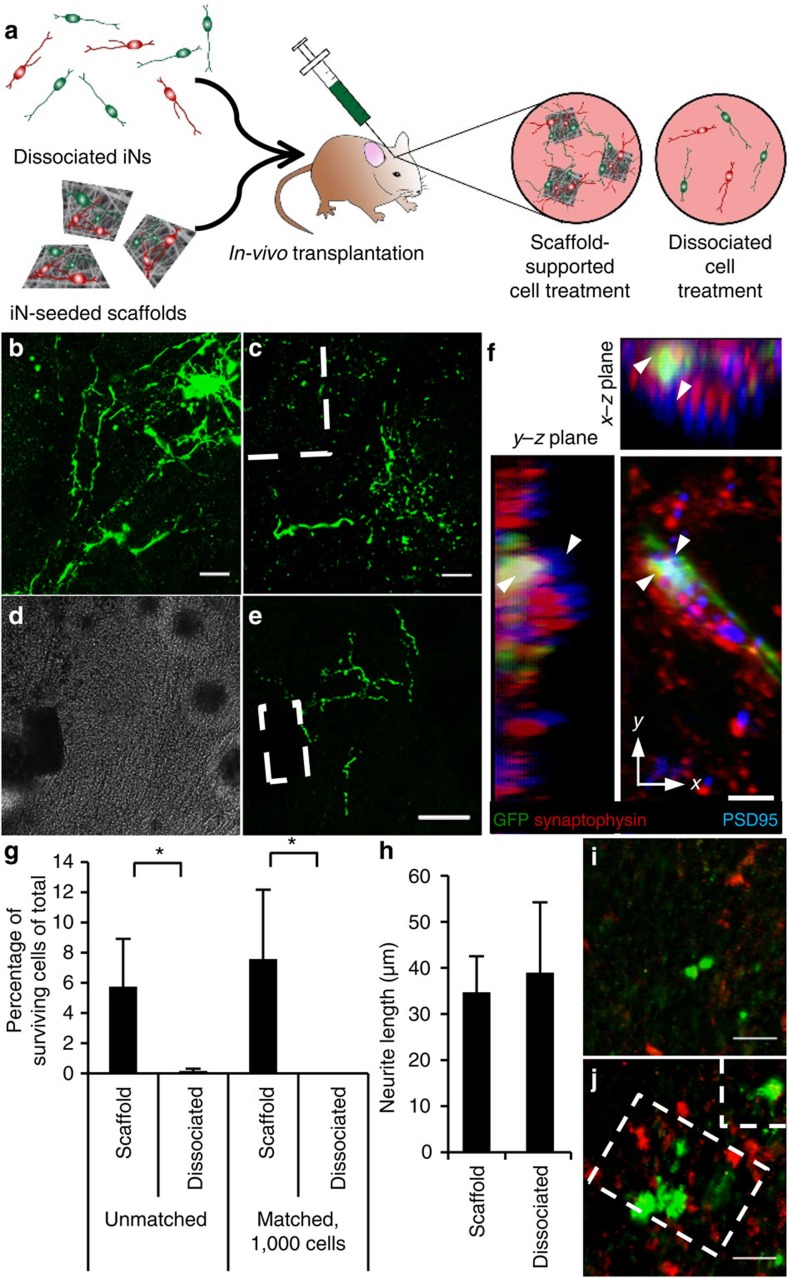
iNs supported by scaffolds support outgrowth and survival *in vivo*. (**a**) iN-seeded scaffolds were injected into mouse striatum and compared with injected dissociated cells. GFP-expressing surviving iNs were found 3 weeks post transplantation in mouse striatum, both for dissociated iNs (**b**) and with iN-seeded microscaffolds (**c**–**e**), located in the vicinity of microscaffolds (indicated with dashed line). Scale bar, 25 μm; 100 μm for **b**,**c** and **d**,**e**, respectively. (**f**) Post-synaptic density protein 95 (PSD-95, blue, indicated using downward-pointing arrows) was detected adjacent to transplanted GFP-labelled iN neurite terminals, which co-localized with synaptophysin (red, with red+green or yellow regions indicated with upward-pointing arrows), suggestive of synaptic integration with host tissue. Scale bar, 5 μm. (**g**) Quantification of surviving cells yielded an average survival rate of 5.74±3.16% out of an average 802±95.8 injected scaffold-seeded iN cells (*n*=3), compared with an average survival rate of 0.15±0.15% out of 100,000 injected dissociated iN cells, or 7.58±4.60% of injected scaffold-seeded iN cells compared with 0±0% out of 1,000 injected dissociated iN cells, for a similar-magnitude number of injected cell comparison (*n*=3). *P<0.05 by one-way ANOVA, all error bars presented as mean±1 s.d. There was no significant difference in neurite length between the two modes of transplanted cells (**h**). GFP-expressing NeuroD1 iNs and RFP-expressing dopamine neurons were preserved in close proximity when co-transplanted on microscaffolds (indicated with dashed line) (**j**), while only some sparse dissociated and transplanted iNs survived (**i**) 1 week post transplantation into mouse striatum. Scale bar, 25 μm.

Next, human iN cell survival was assessed after transplantation of scaffold-supported or dissociated cells into the mouse striatum *in vivo*. Three weeks after transplantation (Fig. 5a), immunocytochemistry of a 1.8 mm × 1.8 mm field including the injection site (Fig. 5b–e) revealed an average survival rate of 5.74±3.16% based on injected scaffold-seeded iN cells, a 38-fold improvement (*P*<0.05, one-way ANOVA) compared with an average survival rate of 0.15±0.15% of 100,000 injected dissociated iN cells (Fig. 5g). The survival rate of dissociated iN cell controls was comparable to that reported by Zhang *et al*.^10^ When magnitude of injected cells were matched at ∼1,000 cells, quantification of surviving cells yielded an average of 7.58±4.60% of injected scaffold-seeded iN cells compared with 0±0% out of 1,000 injected dissociated iN cells. The scaffold-seeded iNs maintained neurite length (35±8 μm) comparable to those of viable, dissociated iNs (39±15 μm) in the experiment when number of injected cells was unmatched. No overt difference in inflammatory response was detected between injection modes, and some ingrowth of host tissue into scaffolds was observed (Supplementary Fig. 8). Surviving transplanted iNs expressed neuronal cell-adhesion molecule CD56, βIII-tubulin and synaptophysin (Fig. 5f, Supplementary Fig. 9). Post-synaptic density protein 95 (PSD95, depicted in blue, with downward-pointing arrows) was detected adjacent or co-localized to transplanted GFP-labelled and synaptophysin-expressing iN neurite terminals, suggestive of synaptic integration with host tissue (Fig. 5f; Supplementary Movie 1). Notably, when microscale scaffolds were used to transplant multiple subtypes of neurons, we observed retention of neurons of distinct specificity in close apposition *in vivo* (Fig. 5i,j). Similar differences in survival were also seen when glutamatergic and dopamine iNs (Supplementary Fig. 10) were co-transplanted either dissociated or on microscaffolds. In all *in vivo* experiments, some human iNs were observed to migrate off scaffolds, and minimal scaffold degradation was observed in the 1–3 weeks post-transplantation.

## Discussion

Cell therapies for treatment of CNS brain injury and disease are challenged by poor cell survival, engraftment and retention after transplantation both into the brain and spinal cord, with cell survival of<1% routinely reported^28^. The objective of this study was to advance a new integrated biomaterials-based paradigm to both rapidly reprogram pluripotent stem cells to neurons and efficiently deliver enriched, organized neuronal networks to the brain. To achieve this, we engineered human iPS cells with the neuronal TF, *NeuroD1*, and demonstrated efficient and rapid neuronal conversion in 3D fibrous substrates fabricated from synthetic polymers that were geometrically tuned to accelerate neuronal maturation and network establishment. Utilizing this system, we report that 3D microfibrous substrates can guide many important characteristics of human iN cells derived from iPS cells that are relevant for *in vivo* applications, including (i) *in vitro* maturation, electrical activity and purification; (ii) neurite outgrowth, survival and electrical activity after transplantation into *ex vivo* brain tissue and (iii) survival and engraftment after transplantation *in vivo* into the striatum. These results highlight the promise of biomaterials-based delivery as a platform for reprogramming and regenerative cell sourcing in the CNS and as a potential model that can be further adapted and evaluated for therapeutic efficacy.

Our primary hypothesis was that microfibrous substrates with thick fibres and interfibre spacing would provide a 3D microniche for rapid, high-purity conversion and accelerated maturation of iPS cell-derived iNs, and that such 3D substrate-supported neuronal networks would exhibit improved levels of retention and engraftment following transplantation into the brain. *In vitro*, thick fibre substrates enhanced human iNs maturation and excitability, while selectively excluding residual undifferentiated iPS cells. Both of these phenomena arise from controlled cell confinement and 3D juxtacrine signalling organization. While the enhanced MAP2 expression in iN cells in thick fibre substrates is consistent with previously reported results^18^,^29^,^30^,^31^, the concurrent increase in excitability indicates active both phenotypic and functional maturation. We propose that the thick fibre substrates drive iN confinement^24^, leading to accelerated maturation due to increased engagement of neural cell-adhesion molecules such as L1 or N-cadherin, which have known roles in neural development^32^,^33^,^34^ and neuritogenesis^29^,^31^. In contrast, the thin fibre substrates fail to support neuronal infiltration and aggregation, resulting in the diminished excitability relative to more 3D, thick fibre substrates.

For effective scaffolds that can form reprogrammed neuronal networks, a key feature is that the proliferation of undifferentiated iPS cells should be suppressed. The geometry we have identified for electrospun scaffolds limits the proportion of undifferentiated iPS cells. Undifferentiated human ES and iPS cells require media supplementation with the rho-associated kinase (ROCK) inhibitor Y-27632 to survive dissociation to single cells^35^. In the absence of Y-27632, the degree to which dissociated iPS cells can re-establish E-cadherin-mediated cell–cell contacts that have been demonstrated to be required for survival and proliferation^36^ is severely diminished when seeded onto electrospun fibrous substrates. Isolated pluripotent stem cells have been observed to form viable colonies largely based on motility-induced aggregation rather than single cell clonal expansion when intercellular distances were less than 6.4 μm (ref. 37). The large surface area, added substrate dimension and constrained migration paths of the electrospun fibres effectively increase intercellular distances, which would limit motility-induced aggregation and could explain the observed increase in differentiation of iPS cells and loss of residual proliferative cells on microfibrous substrates. This reduction in residual undifferentiated iPS cells could be used to supplement other methods for eliminating the risk of teratoma formation *in vivo* due to the presence of undifferentiated cells, however, additional studies are required to assess the tumorigenic potential of these cells once delivered *in vivo*.

After establishing a substrate geometry that accelerated iN maturation, the ability of these substrates to promote engraftment of human iN cells into the CNS was first probed in an *ex vivo* organotypic brain slice model. Delivery of iN cells on microscale scaffolds markedly improved both neurite outgrowth and electrical functionality after transplantation compared with the effects of injected isolated cell suspensions. This suggests that cell retention and survival are enhanced because transplantation of iN cells on microscale scaffolds preserves cell–cell contacts while avoiding the need for dissociation. Dissociation of cells before injection likely leads to increased anoikis^38^,^39^, contributing to diminished engraftment after transplantation. The improvement in electrical activity after transplantation of iN cells in scaffolds is promising, and suggests that these benefits may translate *in vivo*.

Transplantation of iN cells in scaffolds into the mouse striatum showed that the percentage of viable cells after 3 weeks was an order-of-magnitude greater than that relative to injection of isolated single cells. This increased survival is likely indicative of decreased anoikis or apoptosis that otherwise arises from enzymatic disruption of cell–cell and cell–matrix interactions^38^,^39^. These results are promising particularly in light of recent studies investigating the effects of human iPS cell-derived neurons on treating CNS disorders that have shown efficacy *in vivo* even with low levels of cell survival^1^,^10^, suggesting that increased cell survival could lead to amplified levels of efficacy of these cell therapeutics. In addition, host neuron post-synaptic markers were detected adjacent to pre-synaptic marker-expressing transplanted neurons, suggesting that single-factor reprogrammed neurons are capable of functionally integrating into host tissue. Interestingly, some authors have found that extensive lesions can actually enhance neuronal graft survival and integration, as denervated targets may provide a positive stimulatory effect to new neurons^40^,^41^, suggesting that even greater cell survival may be possible when applying this scaffold system in a neurodegenerative disease or injury context.

Finally, the ability for 3D microscale scaffolds to support co-culture and concurrent transplantation of neuronal networks consisting of neurons of multiple subtypes was evaluated. Transplantation of intact networks of mixed subtype neuron populations could be an invaluable tool for treating complex brain disorders or injuries that affect multiple subtypes, or for priming transplanted neurons with pre-established synaptic inputs. For example, attempts for transplanting dopamine neurons derived from fetal brain tissue^42^,^43^ or derived from human iPS cells^1^,^44^ have been made and yield amelioration of Parkinson's Disease; with the paradigm we proposed one can envision a model to graft mini-neurocircuitry composed of excitatory-dopaminergic neurons into the host brain. We hypothesize that proper excitation from the mini-neurocircuitry could produce more profound amelioration of locomotive deficits in Parkinson's Disease.

Induced human neurons offer a rich cell source for a variety of *in vitro* and *in vivo* applications in modelling and treating CNS diseases and injuries. Our prototype epitomizes a biomaterial device for subtype specific neuronal reprogramming and transplantation strategy that encourages transplant survival and engraftment. Similar prototypes could be engineered to apply human neuronal cells of varying subtypes for targeted treatment of a wide range of CNS disorders.

## Methods

### Lentivirus production

The lentiviral constructs rtTA-FUW, Tet-O-FUW *NeuroD1*, Tet-O-FUW *EGFP*, Tet-O-FUW *Ascl1*, Tet-O-FUW *Nurr1*, Tet-O-FUW *Lmx1a*, Tet-O-FUW *Pitx3*, Tet-O-FUW *EN1*, Tet-O-FUW *Foxa2*, and the packaging plasmids pMDL.g/pRRE, pMD.2 (VSVg), and RSV-REV were constructed by the Marius Wernig laboratory at Stanford University. Lentivirus particles were generated by first transfecting 293FT cells by the calcium phosphate precipitation method with each of the three packaging plasmids and one of the lentiviral plasmids in standard 293FT medium, which consists of DMEM supplemented with 10% fetal bovine serum, 1% non-essential amino acids (all from Life Technologies, Carlsbad, CA) and 1% penicillin/streptomycin (Lonza, Walkersville, PA)^45^. Medium is replaced after 24 h and medium containing viral particles is collected at 48 and 72 h. Viral particles are concentrated 200 × by ultracentrifugation at 25,000 r.p.m. for 2 h at 4 °C, and stored at −80 °C until use.

### iPS cell culture and neuronal conversion

Human iPS cells were obtained from the Rutgers University RUCDR Infinite Biologics. iPS cells were derived from human foreskin fibroblasts by retroviral infection with Oct-4, Sox2, Klf4 and c-Myc^46^. Human iPS cells were cultured on Matrigel-treated dishes in mTeSR-1 (ref. 24). iPS cells were passaged with dispase every 5–7 days with manual removal of spontaneously differentiated areas with fire-polished glass pipets. iPS cells from passage 15–35 were used in these studies. iPS cells were passaged as single cells with Accutase (Stem Cell Technologies) plated at high density (8 × 10^4^ cells per cm^2^) in mTeSR-1 supplemented with 5 μM Y-27632 (R&D Systems, Minneapolis, MN)^35^. The following day, medium was replaced with mTeSR-1 with 5 μM Y-27632 and 2 μM polybrene (Sigma-Aldrich, St Louis, MO) and concentrated virus was added (rtTA-FUW, Tet-O-FUW *NeuroD1*, and in some experiments, Tet-O-FUW *EGFP*). The following day, medium was replaced with mTeSR-1 and cells were returned to normal culture for up to eight additional passages. To induce neuronal conversion, infected iPSCs (termed iPSC-RNs) were passaged with Accutase and Y-27632, and the following day switched to an N2-based conversion medium (N2M) consisting of DMEM/F12, 2 mM L-glutamine, 1 × N2 supplement, 1 × non-essential amino acids, 1% penicillin/streptomycin and 2 μg ml^−1^ doxycycline, which induces expression of the *NeuroD1* and *EGFP* constructs. Medium was replaced daily, and cells were replated on day 3–5 onto 2D or 3D substrates. One day after replating, medium was switched to NBM, consisting of neurobasal medium, 2 mM L-glutamine, 1 × B27 supplement without Vitamin A (Life Technologies), 0.2 mM ascorbic acid, 1 μM cAMP (Sigma-Aldrich), 2 μg ml^−1^ doxycycline and 10 ng ml^−1^ each of brain-derived neurotrophic factor, Glial cell-line derived neurotrophic factor and neurotrophin-3 (Peprotech). Medium was replaced every 2–3 days for the duration of the experiments.

To generate dopamine iNs for co-culture studies, 2.5 × 10^5^ cells per six-well plate were plated, and the following day medium was replaced with mTeSR-1 with 5 μM Y-27632, 2 μM polybrene, and concentrated virus was added (rtTA-FUW, Ascl1, Nurr1, Lmx1a and, in some experiments, Tet-O-FUW *RFP*). After three days, medium was replaced with Neurobasal media (Life Technologies) and concentrated virus (Pitx3, EN1, Foxa2). Four days after initial infection, the dopamine iNs would be co-seeded onto microscale scaffolds.

### Electrospun fibrous substrate fabrication

Fibrous substrates and 2D polymer film controls were fabricated from tyrosine-derived polycarbonate pDTEc via electrospinning and spin-coating, respectively^24^. These polymers come from a combinatorial library of polymers with tunable hydrophobicity and degradation properties. pDTEc was dissolved in 1,1,1,3,3,3-Hexafluoro-2-propanol (Sigma-Aldrich) at 9%, or 18% weight by volume for thin and thick fibre configurations respectively. For the 9% weight by volume solution, 5% N,N-dimethylformamide was added to prevent beading of fibres. Polymer solutions were electrospun from a +18 kV spinneret to a −6 kV flat plate collector at 3 ml h^−1^ over a distance of 30 cm, resulting in fibre mats 250–500-μm thick. Surface morphology was observed on an AMRAY 1830 I scanning electron microscope and fibre diameter was quantified by measuring 100 individual fibres from each fibrous substrate using NIH-ImageJ software ( http://rsb.info.nih.gov/ij/). Quantitative data are presented as mean±95% confidence interval. Control 2D polymer films were prepared using a 1% polymer solution dissolved in tetrahydrofuran, which was then spin-coated onto glass coverslips at 4,000 r.p.m. for 30 s (ref. 47). For *in vitro* studies, electrospun fibres were sterilized by ultraviolet treatment, oxygen plasma treated for hydration, and adsorbed with 10 μg ml^−1^ poly-D-lysine in HEPES buffer (pH 8.4) and 4 μg ml^−1^ laminin in PBS before seeding dissociated iNs 4 days after initiating neuronal conversion with N2M+dox.

### Microscale scaffold preparation and neuronal seeding

Microscale fibrous scaffolds were prepared by cutting oxygen plasma treated and hydrated electrospun fibre mats into 100 μm squares with a McIlwain tissue chopper (Vibratome), and sterilized and coated with poly-D-lysine and laminin as described above. On day 4 of neuronal conversion, iN cells were dissociated with Accutase and resuspended in N2M with dox at 5 million cells per ml. The scaffolds and iN cell suspension were mixed in a 48-well non-TCPS plate for 1 h with occasional gentle agitation, followed by three washes to remove unattached cells. Scaffolds containing iN cells were cultured for 1 day in N2M, then cultured in NBM until used for *ex vivo* or *in vivo* experiments. For experiments examining co-cultures of dopamine iNs and NeuroD1 iNs, dissociated dopamine and NeuroD1 iNs were dissociated, resuspended and combined 1:1 ratios to a concentration of 5 million total cells per ml.

### *Ex vivo* slice preparation and scaffold transplantation

Organotypic mouse brain slice cultures were prepared by isolating the cortico-striatal area of 6-day-old C57BL/6 mouse brains and cutting into 300 μm slices using a vibratome (Leica Microsystems) in ice-cold sucrose solution (204 mM sucrose, 26.2 mM NaHCO_3_, 11 mM glucose, 2.5 mM KCl, 2 mM MgSO_4_, 1 mM NaH_2_PO_4_, 0.5 mM CaCl_2_ saturated with 95% O_2_ and 5% CO_2_) (ref. 48). Slices were transferred onto Millicell organotypic cell culture inserts (Millipore) in a six-well tissue-culture plate and cultured in Neurobasal A medium (Life Technologies) supplemented with 1 μg ml^−1^ insulin, 0.5 mM ascorbic acid, 25% horse serum, and 1% penicillin/streptomycin. Mouse brain slices were cultured for 2 days before iN cell transplantation. Two days after preparing brain slices, dissociated and scaffold-seeded GFP^+^ iN cells (day 6 post-conversion) were transplanted onto the surface of the cortico-striatal slices using a pipette. Medium was changed every 2–3 days and slices were fixed in 4% paraformaldehyde after 7 days for immunohistochemistry to quantify neurite outgrowth, or cultured 14 days before electrophysiology studies.

### *In vivo* cell injection and scaffold transplantation

All animal experiments were carried out according to the Rutgers University Policy on Animal Welfare and were approved by the Institutional Animal Care and Use Committee (IACUC) at Rutgers University Robert Wood Johnson Medical School. Male, 5-week-old *NOD-SCID IL2Rγc* null mice (20–35 g; Jackson Laboratory) were anaesthetised with isoflurane (induction at 4% and maintained at 0.5–1% inhalation) and injected with a total volume of 10 μl, containing either 1 × 10^3^, 1 × 10^5^ dissociated GFP^+^ iN cells or ∼10 scaffolds seeded with GFP^+^ iN cells (∼85 cells per scaffold) at day 6 after initiating neuronal conversion, resuspended in ice-cold MEM (Life Technologies). Cells or cells on scaffolds were injected stereotactically into the striatum using a 100 μl gastight Hamilton syringe and 21G Hamilton needle. Bilateral injections were made at the following coordinates (in mm): AP, 0.5 (from bregma); ML, −2.0; DV, −3.0 (from dura). Mice were killed and processed for immunohistochemistry 3 weeks after transplantation for quantification of human iN cells within the graft by visualization of the human-specific nuclei antibody HuNu (Millipore) GFP, and RFP in the case of multiple-subtype co-transplantation (see Supplementary Table 1 for a detailed list of primary antibodies). Neurite outgrowth was quantified by measurement of GFP^+^ neurites.

### Electrophysiology

Electrophysiolocial recordings were performed at prescribed time intervals from iN cells in 2D or 3D configurations in whole-cell mode using a Multiclamp 700B amplifier^12^,^45^. The bath solution contained 140 mM NaCl, 5 mM KCl, 2 mM MgCl_1_, 2 mM CaCl_2_, 10 mM HEPES and 10 mM glucose, at pH 7.4. The pipette solution for whole-cell voltage-dependent current recordings and for current-clamp experiments contained 10 mM KCl, 123 mM K-gluconate, 1 mM MgCl_2_, 10 mM HEPES, 4 mM glucose, 1 mM EGTA, 0.1 mM CaCl_2_, 1 mM K_2_ATP, 0.2 mM Na_4_GTP, pH 7.2. Membrane resting potentials were kept in the range of −65 to −70 mV, with step currents injected to elicit action potentials.

### Immunocytochemistry

Neuronal phenotypes were characterized by immunocytochemistry for 2D and 3D substrates. Cells were fixed with 4% paraformaldehyde for 30 min at room temperature, followed by three washes with PBS. Cells were simultaneously blocked and permeabilized in blocking buffer consisting of PBS supplemented with 5% normal goat serum (MP Biomedicals), 1% bovine serum albumin (Sigma-Aldrich), and 0.1% Triton X-100 (Sigma-Aldrich) for 1 h at room temperature. Primary antibodies were incubated in blocking buffer at 4 °C overnight, followed by three 15-min PBS washes. Fluorophore-conjugated secondary antibodies (Alexa Fluor 488, 594, or 647, Life Technologies) were incubated in blocking buffer for 1 h at room temperature, followed by three 15-min washes and at least one wash of 1–2 h. Samples were counter-stained with 4′,6-Diamidino-2-phenylindole dihydrochloride (Sigma-Aldrich) to visualize nuclei and mounted with Prolong Gold Anti-Fade reagent (Life Technologies) before imaging. Samples were imaged on a Leica SP2 laser scanning confocal microscope using 10 × dry or 63 × immersion objectives. Images of cells on 3D electrospun fibres are presented as maximum intensity projections of z-stacks, unless otherwise stated. Image quantification was performed via custom ImageJ macros and manual counting. A complete listing of the primary antibodies used in this study can be found in Supplementary Table 1.

### Calcium imaging

iN cells were labelled with 3 μM Fluo-4 AM calcium indicator dye for 30 min at room temperature in extracellular bath solution (147 μM NaCl, 5 μM KCl, 2 mM CaCl_2_, 2 mM MgCl_2_, 10 mM HEPES and 10 uM Glucose, pH 7.4 and osmolarity 290–300 mOsm) containing 0.02% pluronic F-127. After incubation, cells were rinsed twice with bath solution and incubated for an additional 30 min at room temperature to allow complete de-esterification of the Fluo-4 AM dye before imaging. Time-lapse imaging was performed on a Leica SP2 confocal microscope at a resolution of 512 × 512 pixels and a time resolution of 1 Hz. Image analysis was performed using custom ImageJ macros and MATLAB routines for semi-automated ROI selection and peak detection to quantify the fraction of cells that were electrically active. Cells were considered to be electrically active if they showed a spike in fluorescence following application of an electrical stimulus. The electrical stimulus was applied using a function generator (Global Specialties) and consisted of a 5 s stimulus at 7.5 V cm^−1^ with 5 ms square pulses at 40 Hz.

### Quantitative real-time polymerase chain reaction

Total RNA was extracted from iPS cells cultured in 2D or 3D using the RNEasy kit (Qiagen, Valencia, CA) according to the manufacturer's instructions, including treatment with RNase-free DNase to remove genomic DNA. A high-capacity cDNA reverse transcription kit (Applied Biosystems, Foster City, CA) containing random primers was used to reverse transcribe 200 ng total RNA from each sample to cDNA. Taqman gene 194 expression master mix and Taqman gene-expression assays (Applied Biosystems) were used for template amplification of 10 ng cDNA per reaction. Quantitative real-time polymerase chain reaction (qRT–PCR) was carried out on a 7500 Fast Real-Time PCR instrument (Applied Biosystems). Relative gene expression was calculated using the ΔΔCT method, normalizing to GAPDH as the endogenous control and undifferentiated IPS cells in standard 2D culture (on Matrigel-coated dishes in mTeSR media) after 7 days as the reference sample. Taqman gene-expression assays were used in these studies, listed in Supplementary Table 2.

### Statistical analysis

All data are presented as mean±s.d. Statistical significance is evaluated by single-factor ANOVA and a Tukey's *post hoc* test, with *P*<0.05 considered statistically significant.

## Additional information

**How to cite this article**: Carlson, A. L. *et al*. Generation and transplantation of reprogrammed human neurons in the brain using 3D microtopographic scaffolds. *Nat. Commun.* 7:10862 doi: 10.1038/ncomms10862 (2016).

## Supplementary Material

Supplementary Information Supplementary Figures 1-10 and Supplementary Tables 1-2

Supplementary Movie 1 Post synaptic density protein 95 (PSD-95) (blue) was detected adjacent to transplanted GFP-labeled iN neurite terminals (green), which colocalized with synaptophysin (red), suggestive of synaptic integration with host tissue. Scale bar = 5 μm.
