# Supplementary material for: Generation and transplantation of reprogrammed human neurons in the brain using 3D microtopographic scaffolds
Source: Nat Commun. 2016 Mar 17;7:10862. doi: 10.1038/ncomms10862 (PMC4800432; doi:10.1038/ncomms10862)
Supplement: Supplementary Information — Supplementary Figures 1-10 and Supplementary Tables 1-2 [file ncomms10862-s1.pdf]

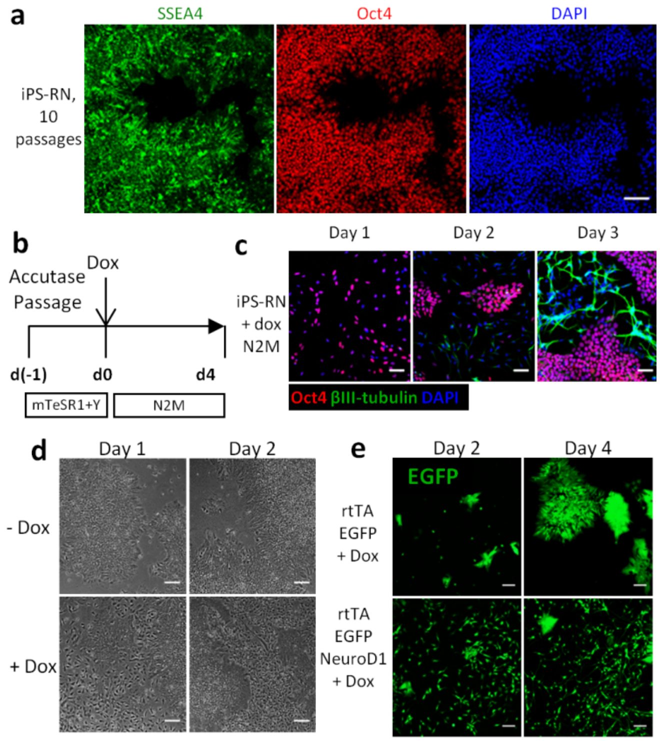

Supplementary Figure 1: Derivation and characterization of RN-iPS cell lines. (a) RN-iPS cells maintain expression of pluripotency markers OCT4 and SSEA4 after 10 passages in mTeSR-1 medium. (b) Schematic of plating and doxycycline (dox) addition for generation of iNs from RN-iPS cells. (c) Many RN-iPS cells lose Oct4 and gain βIII-tubulin expression 3 days after dox addition in N2M. (d) Bright field images after 1 or 2 days of dox treatment reveal that in the absence of dox, cells retain a characteristic undifferentiated iPS cell morphology, where in the presence of dox, cells around the periphery of colonies begin to take on an early neuronal cell morphology. (e) iPS cells infected with rtTA and EGFP show EGFP expression 2 and 4 days after dox addition, but maintain an undifferentiated morphology. iPS cells infected with rtTA, EGFP, and NeuroD1 also show EGFP expression 2 and 4 days after dox addition, but also show a distinct conversion to progressively maturing neuronal morphologies. Scale bar = 100μm (a, d-e), 50 μm (c).

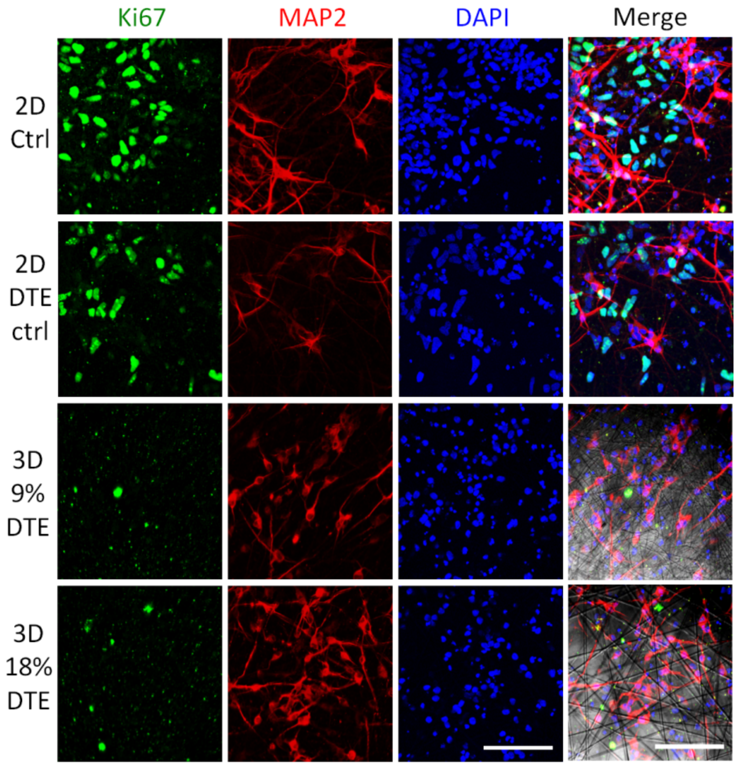

Supplementary Figure 2: Comparison of neuronal selection and maturation in 2D and 3D substrates. iN populations robustly express microtubule-associated protein 2 (MAP2) in 2D and 3D conditions, while populations of unconverted, proliferative Ki67-expressing iPS cells persist in iN populations plated in 2D conditions. Scale bar = 100 μm.

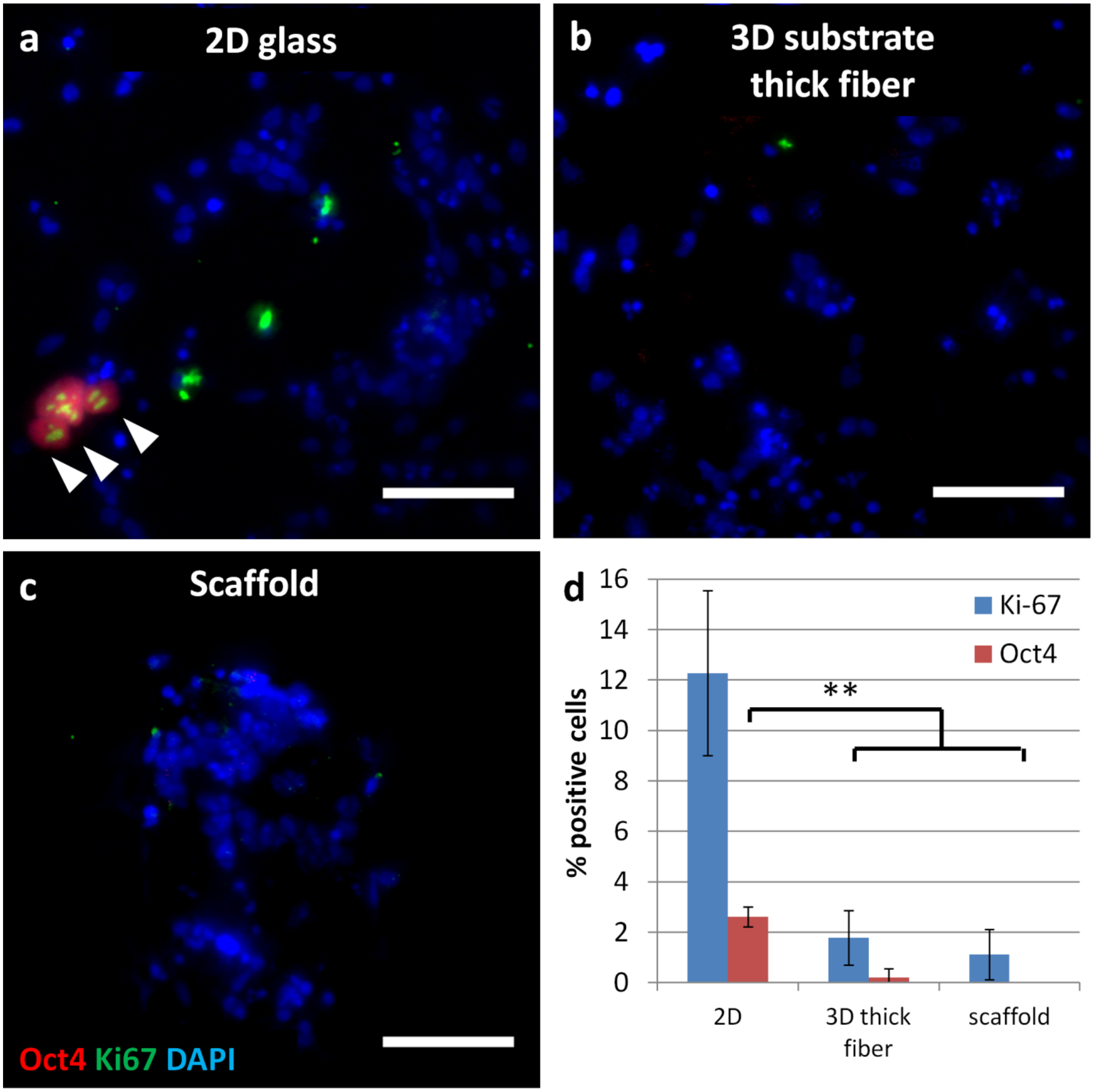

Supplementary Figure 3: Characterization of unconverted induced neuronal cells. (a) iNs replated onto 2D glass surfaces after initiating reprogramming contained populations of Ki67+ cells (green), a subpopulation of Ki-67+ cells were also Oct4+ (red). iNs replated onto thick fiber 3D substrates (b) or scaffolds (c) similarly had significantly fewer of both Ki-67+ and Oct4+ cells, **P<0.01 by 1-way ANOVA, , all error bars presented as mean ± 1 standard deviation.

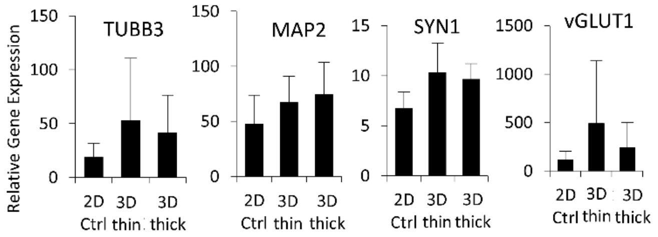

Supplementary Figure 4: qRT-PCR analysis of induced neuron gene expression. Analysis of gene expression profiles reveals similar gene expression profiles between the three substrates, all error bars presented as mean ± 1 standard deviation.

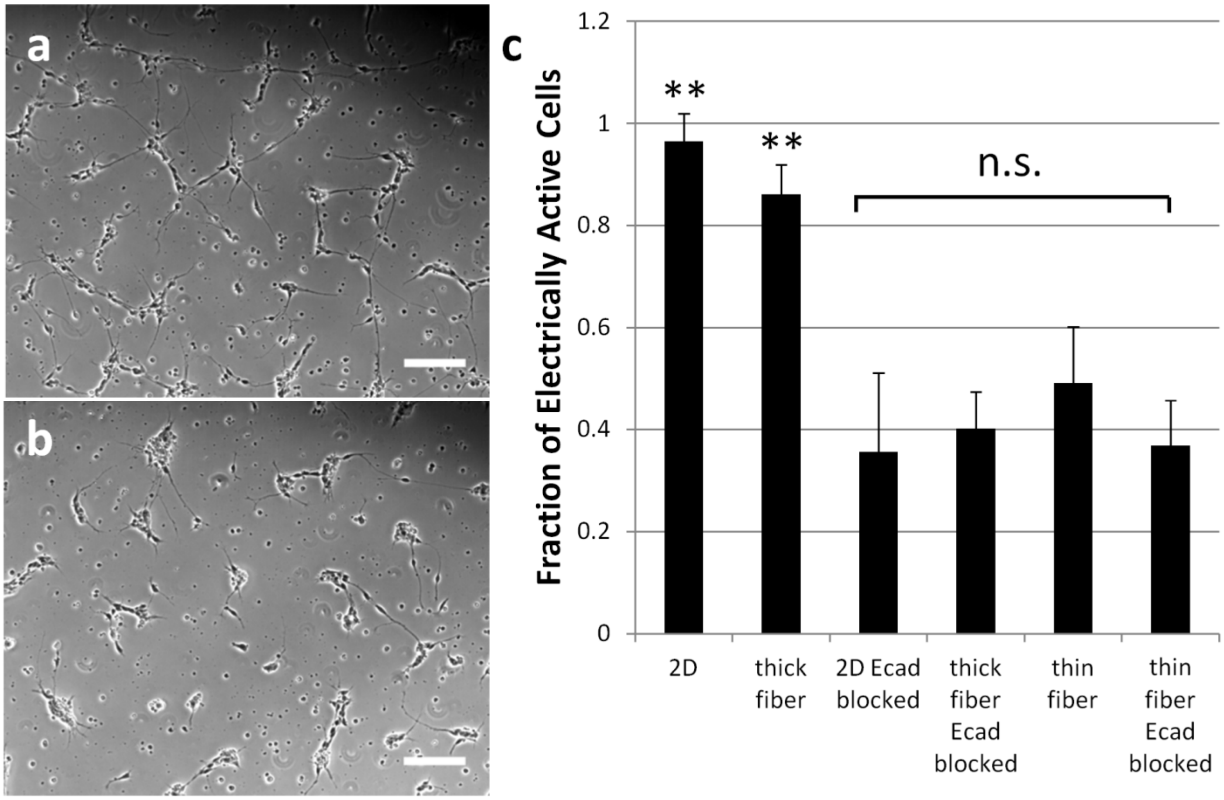

Supplementary Figure 5: Fiber architecture controls neuronal development via cell-cell contact. iN populations were incubated with 200 μg mL^{-1} E-cadherin antibody for 15 minutes prior to seeding onto substrates, either 2D or thick and thin fiber 3D substrates. Compared to uninhibited iN cells in 2D (a), iN cells with inhibited E-cadherin dependent cell-cell contact displayed reduced outgrowth in 2D (b). Inhibited cell-cell contact resulted in decreased activity measured by calcium imaging for iNs on 2-D and thick fiber substrates, but did not decrease activity in thin fiber substrates (c). **$P<0.01$ by 1-way ANOVA relative to respective Ecad blocked conditions. n.s. = not significant. Scale bar = 150 μm, all error bars presented as mean ± 1 standard deviation.

.

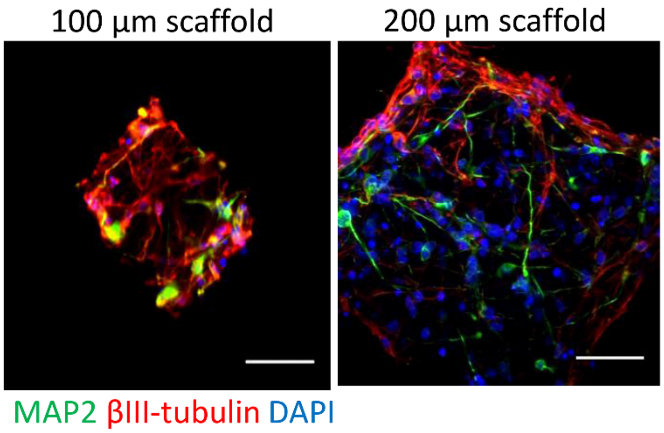

Supplementary Figure 6: Characterization of neuronal maturation on microscale scaffolds. Immunocytochemistry for pan-neuronal markers microtubule-associated protein 2 (MAP2) and βIII-tubulin in iNs after 4 days in 100 and 200 µm scaffold. Scale bar: 50 µm.

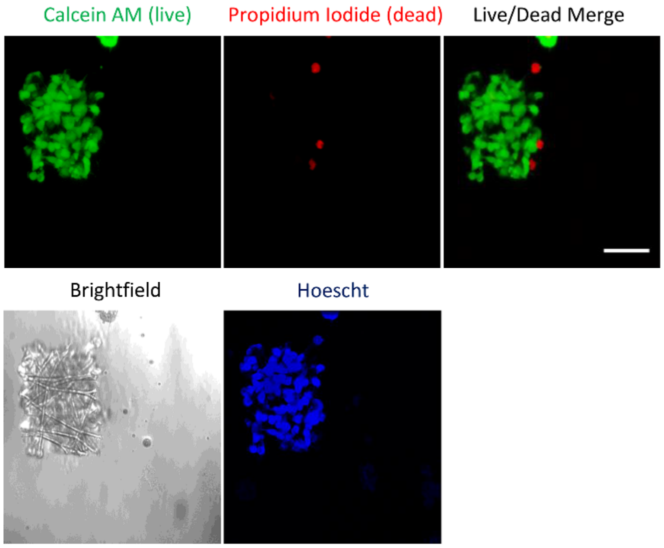

Supplementary Figure 7: Live/dead imaging in a 100 µm scaffold. On average, there were 82.6 ± 13.1 live cells per scaffold. Scale bar: 50 µm.

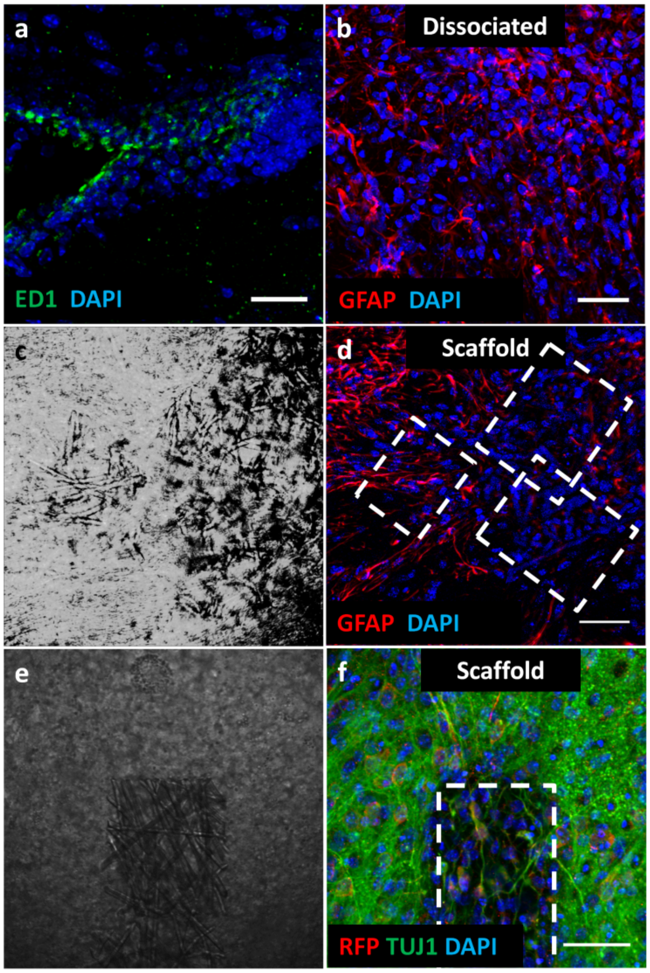

Supplementary Figure 8: Host response to transplanted scaffolds. (a) Activated microglia expressing ED1 or CD68 were seen in injection sites in mice receiving both dissociated and scaffold-supported iNs. Astrocytes, expressing glial fibrillary acidic protein (GFAP) were seen near the injection site in both mice receiving dissociated (b) and scaffold-supported iNs (c-d). Some degree of host tissue ingrowth was observed near transplanted scaffolds with no iNs nearby, visualized using brightfield (e) and immunostaining for βIII-tubulin and RFP, to detect fluorolabeled transplanted cells (f). Scale bars = 50 μm.

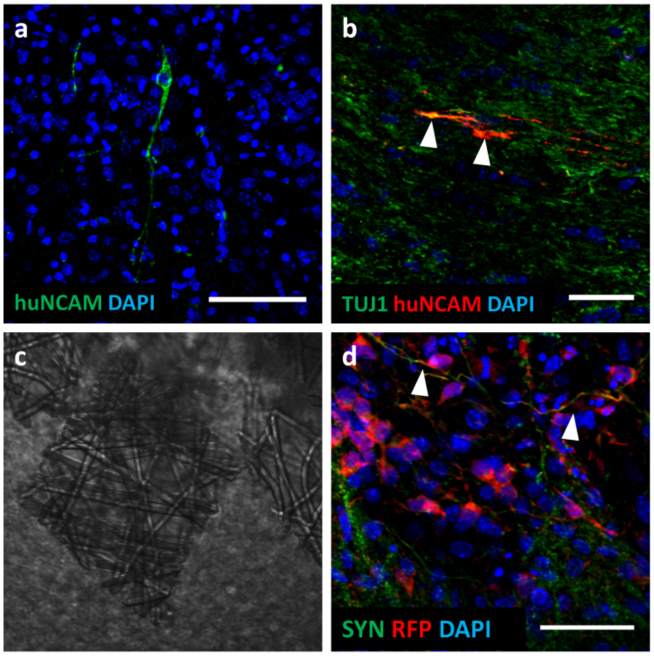

Supplementary Figure 9: Characterization of transplanted iNs. (a) 3 weeks post-transplantation into mouse striatum, iNs expressed neural cell adhesion molecule CD56, detected with a human specific antibody. Scale bar = 100 µm. (b) Cells that positively expressed human specific neural cell adhesion (red) molecule also expressed β-III tubulin (green), indicated by arrowheads pointing to yellow colored neurites. Scale bar = 50 µm. Scaffold supported RFP-labelled iNs (red), located near brightfield scaffolds (c) were found to express synaptophysin (green) (d), two such red-colored neurites with distinct green regions are indicated with arrowheads, scale bar = 50 µm.

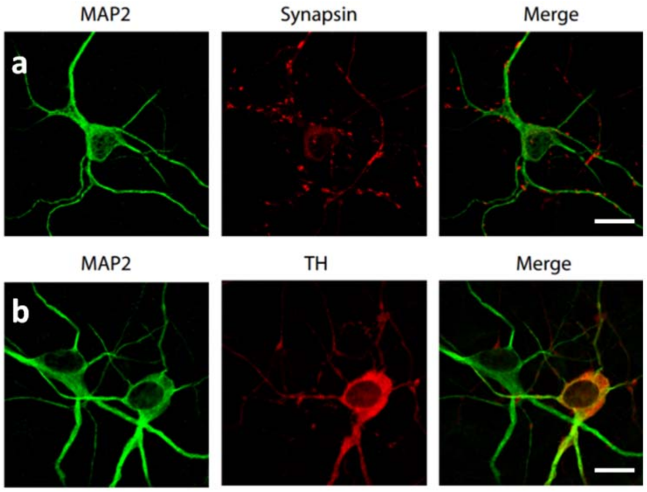

Supplementary Figure 10: Induced DA neurons stain positively for mature neuronal markers and dopamine markers. (a) iDA neurons express microtubule-associated protein 2 (MAP2) and Synapsin. (b) iDA neurons express MAP2 and tyrosine hydroxylase (TH). Scale bars = 20 µm.

Supplementary Table 1: Antibodies used for immunocytochemistry.

Antibody	Species	Isotype	Dilution	Company	Product #
βIII-tubulin	mouse	IgG 2a	1:1000	Covance	MMS-435P
Map2	mouse	IgG 1	1:500	BD Biosciences	556320
Synaptophysin	rabbit	IgG	1:250	Millipore	04-1019
Oct4	mouse	IgG1	1:1000	Millipore	MAB4401
TH	mouse	IgG 2a	1:100-1:500	Millipore	MAB5280
GFP	chicken	IgY	1:1000	abcam	ab13970
RFP	rabbit	IgG	1:100	Abcam	ab62341
huNCAM	mouse	IgG1	1:100	Santa Cruz	sc-106
HuNu	mouse	IgG1	1:100	Millipore	MAB1281
ki67	rabbit	IgG	1:50-1:100	abcam	ab15580
E-cadherin	mouse	IgG1	1:10	Invitrogen	13-1700
vgat	mouse	IgG3	1:1000	synaptic systems	131 001
vglut	rabbit	IgG	1:1000	synaptic systems	135 302
PSD-95	mouse	IgG2ak	1:500	Millipore	MABN68

Supplementary Table 2: Taqman gene expression assays used for qRT-PCR.

Gene	Taqman Gene Expression Assay
GAPDH	Cat#: 4326317E
TUBB3	Hs00801390_s1
MAP2	Hs00258900_m1
SYN1	Hs00199577_m1
SLC17A7	Hs00220404_m1
